# Ribosome-Associated Quality Control Mediated by Rqc2 Contributes to the Lytic Cycle and Stage Conversion of *Toxoplasma gondii*

**DOI:** 10.3390/microorganisms13092041

**Published:** 2025-08-31

**Authors:** Yuxue Li, Keqin Huang, Honglin Jia, Xu Gao, Huanping Guo

**Affiliations:** 1 State Key Laboratory for Animal Disease Control and Prevention, Harbin Veterinary Research Institute, Chinese Academy of Agricultural Sciences, Harbin 150069, Chinajiahonglin@caas.cn (H.J.); 2 Laboratory for Preventive Veterinary Medicine, Department of Veterinary Medicine, College of Agricultural, Yanbian University, Yanji 133000, China

**Keywords:** bradyzoite, NFACT, tachyzoite, TgRqc2, *Toxoplasma gondii*

## Abstract

The conversion from fast-growing tachyzoites to slow-growing bradyzoites is the key factor in establishing the chronic infection and long-term persistence of *Toxoplasma gondii*. Environmental stressors, such as amino acid starvation and alkaline medium, can trigger the transformation of tachyzoites into bradyzoites. Under such stress conditions, ribosomes slow down, potentially leading to stalling, and ribosomal collisions typically activate ribosome-associated quality control (RQC) pathways. In this study, we investigated the role of *T. gondii* ribosome quality control complex subunit 2 (TgRqc2), which contains both NFACT and coiled-coil domains, in the parasite’s survival and stage conversion. NFACT represents the “domain” found in the central players involved in RQC, human NEMF and its orthologs FbpA (known as RqcH), Caliban, and Tae2 (known as Rqc2). Phylogenetic analyses revealed that TgRqc2 formed a distinct clade with its orthologs in apicomplexan parasites. The deletion of TgRqc2 impaired *T. gondii’s* invasion and replication. The Rqc2-knockout strain showed defects in plaque formation and bradyzoite development. Our findings demonstrate that TgRqc2 is essential for *T. gondii’s* lytic cycle and the conversion of tachyzoites into bradyzoites. RNA-seq analysis further showed that the depletion of TgRqc2 significantly disrupted global transcriptional activity. However, the detailed molecular mechanisms involved remain to be elucidated. In conclusion, our results proved valuable insights that may aid in the development of therapeutic strategies to prevent chronic infection.

## 1. Introduction

*Toxoplasma gondii* is an opportunistic intracellular parasite capable of infecting nearly all warm-blooded animals, including humans, and is the causative agent of toxoplasmosis. The life cycle of *T. gondii* is complex, involving both sexual/asexual reproduction in the definitive host and exclusively asexual reproduction in intermediate hosts [1]. The parasite exists in three infective stages: tachyzoites (in groups or clones), bradyzoites (in tissue cysts), and sporozoites (in oocysts) [1]. Tachyzoites disseminate throughout the body, causing acute infection before differentiating into bradyzoite-containing cysts, which establish a lifelong latent infection [2]. While most infections are controlled by the host’s immune response, the reactivation of latent infections can occur during periods of immunosuppression. The active replication of tachyzoites can lead to severe and potentially fatal disease in immunocompromised individuals. Given the central role of stage transition in *Toxoplasma* pathogenesis and disease progression, understanding the complex mechanisms involved in stage conversion remains a critical and urgent priority.

Tachyzoite–bradyzoite interconversion involves an extensive reprogramming of gene expression, with multiple transcription factors and chromatin remodeling complexes playing essential roles in this regulatory process [3,4,5]. In comparison to transcriptional control, the translational regulation of available mRNA provides a more immediate, yet crucial, influence on the composition and abundance of the proteome in response to environmental stress [6]. Translational control not only affects cellular proliferation and growth but also modulates a wide range of physiological processes [7,8]. In *T. gondii*, regulated translation is particularly important for stage conversion and involves the action of translation initiation factors [9,10]. During translation, ribosome stalling can occur for various reasons. Notably, certain *T. gondii* strains, such as typeⅡ strains ME49 and PLK, can be induced to transition into the bradyzoite stage under alkaline medium in vitro [11]. Like other established methods of inducing stage conversion—such as exposure to acidic environments or heat shock—these treatments appear to depend on imposing stress on the parasites [3]. Consequently, stage conversion is likely accompanied by ribosome collisions, which may arise due to slowed ribosomal activity under such stress conditions [12].

Errors can occur during gene expression, potentially leading to the synthesis of proteins with multiple forms of variation. One notable source of abnormal proteins that has garnered significant attention in the past decade is ribosome stalling during translation. Abnormal proteins can cause deleterious effects on cell growth and therefore must be eliminated. This function is accomplished by a specialized monitoring mechanism, which is known as the ribosome-associated quality control (RQC) [13]. Ribosome collisions typically initiate the RQC response [14,15]. The RQC pathway involves the participation of the nuclear export mediator factor (NEMF) in humans and its orthologs, yeast Rqc2 and bacterial RqcH, which recruit the E3 ligase Listerin to ubiquitinate ribosome-stalled nascent polypeptides via lysine residue for degradation [16,17]. The core structure of NEMF and its orthologs consists of NFACT and coiled-coil domains. NFACT refers to a “domain’ found in key RQC components, including human NEMF and its orthologs, FbpA (known as RqcH), Caliban, and Tae2 (known as Rqc2) [16]. RQC defects impair cellular fitness and cause abnormal cellular processes. Although our understanding of RQC’s mechanism in cellular function is expanding, little is known about the specific components of RQC and its role in the growth and development of *T. gondii*.

In this study, we identified and characterized an ortholog of Rqc2 in *T. gondii,* which we named TgRqc2. TgRqc2 contains both an NAFCT RNA-binding and an NFACT-C domain which serves as the central region of the protein involved in regulating translation through the RQC pathway. Our findings demonstrate that TgRqc2 is essential for *T. gondii’s* lytic cycle and conversion to bradyzoite. RNA-seq analysis revealed that the depletion of TgRqc2 significantly disrupted global transcriptional activity across the genome.

## 2. Materials and Methods

### 2.1. Animal Studies and Ethical Approval

Seven-week-old female BALB/c mice were purchased from Liaoning Changsheng Biotechnology Co., LTD. The 12 mice were randomly divided into two groups and housed in cages with 6 mice per cage under optimal conditions, with a temperature of 24 + 3 °C and relative humidity of about 50%. After one week to acclimatize to the environment, each mouse in the control group was infected with 500 tachyzoites of the wild-type strain diluted in 200 μL phosphate buffered saline (PBS). Similarly, each mouse in the experimental group was infected with 500 tachyzoites of the Rqc2-knockout strain. After one month of monitoring, the surviving mice were executed after anesthesia, and their brains were collected and used for the isolation of cyst tissue. All animal experiments used in this research were approved by the Ethical Committee of Harbin Veterinary Research Institute, Chinese Academy of Agricultural Sciences (250425-01-GR). The housing of mice were carried out in strict accordance with the regulation of the Animal Ethics Procedures and Guidelines of the Peoples’ Republic of China.

### 2.2. Sequences Analysis and Phylogeny

The protein sequences of *T. gondii* [TGME49_214090], *Neospora caninum* [F0VKV7], Besnoitia besnoiti [A0A2A9MLD6], *Plasmodium falciparum* [A0A144A0J9], *Saccharomyces cerevisiae* [Q12532], *Channa striata* [A0AA88SB22], *Schizosaccharomyces pombe* [Q9USN8], *Mus musculus* [Q8CCP0], *Oryzias javanicus* [A0A3S2MEM9], *Homo sapiens* [Q9BQ70] [O60524], *Drosophila melanogaster* [Q9VBX1], *Triticum aestivum* [A0A3B6MNN1], *Streptococcus pneumoniae* [Q8DQ36], *Oryza sativa subsp. japonica* [Q2QNI2], and *Staphylococcus aureus* [G3CGC6] were extracted from ToxoDB (https://toxodb.org/toxo/app)(accessed on 20 June 2024) and Uniprot (https://uniprot.org) (accessed on 20 June 2024). Alignment and phylogenetic reconstructions were carried out using the “build” function of ETE3 version 3.1.3 [18] as implemented on GenomeNet (https://www.genome.jp/tools/ete/)(accessed on 20 June 2024). Multiple sequence alignment was provided by the user. The maximum likelihood tree was inferred using RAxML v8.2.11 with the PROTGAMMAJTT substitution model and default parameters [19]. Branch support was assessed using 100 bootstrap replicates. Domain alignment was performed in MEGA version 11.0.13 using ClustaIW (https://www.genome.jp/tools-bin/clustalw) (accessed on 14 June 2025). A neighbor-joining tree was generated and visualized in MEGA with the bootstrap method (1000 replications) as a test of phylogeny, applying the remaining default parameters.

### 2.3. Parasite Strains and Culturing

The ME49 strain (a donation kindly provided by Professor Yurong Yang from Henan Agricultural University) and the Rqc2-knockout strain were cultured on the HFF cell line (ATCC CRL-4001). The cells were maintained in Dulbecco’s Modified Eagle’s Medium (DMEM, Sigma-Aldrich, StLouis, MO, USA) supplemented with 20% (*v*/*v*) Medium 199 Earle’s Salts (ThermoFisher Scientific, Grand Island, NY, USA), 10% (*v*/*v*) fetal bovine serum (Clark, Virginia, USA), and 2% penicillin/streptavidin.

### 2.4. Construction of Plasmids

The guide RNA sequences specific to TgRqc2 (N-gRNA: ACTGGACGTCCGGGCTCTCG; C-gRNA: GAAAAAGACCGTCACAGCTG) were designed by the CRISPR guide design tool (http://grna.ctegd.uga.edu/)(accessed on june 26, 2024)and cloned into the *Eco*RⅠ site of the Cas9 plasmids to construct the CRISPR/Cas9 plasmid. The homology arms of TgRqc2 were amplified and introduced into the PGEM-18T vector using the primers (TgRqc2-UP-HR-F: GTTCCGCTTCTTTTGTGTCT; TgRqc2-UP-HR-R: TCTCGCGCAGCAGACAAGAA; TgRqc2-DOWN-HR-F: GTCTGCTGCGCGAGAACATGCAGCAAAGTGTCTCA; TgRqc2-DOWN-HR-R: ATATATACATTCACACATAT). All PCRs in this study were performed using TaKaRa Ex Taq (Takara, Kusatsu, Shiga, Japan ) or KOD-Plus-Neo (Toyobo, Osaka, Japan).

### 2.5. Transfection and Selection of Parasites

To obtain a single clone strain with the target gene knocked out, tachyzoites (10^7^) were harvested and placed in a 2 mm gap cuvette (Bio-Rad, Herculaneum, CA, USA). Then, electroporation was performed using the standard procedures as previously reported [20]. In brief, the standard electroporation procedures were performed at 25 μF, 50 Ω, 1500 V, and 2 mm. Transgenic tachyzoites were cultured in Vero cells for 24 h and then sorted by flow cytometry according to the expressed GFP protein. Sorted parasites were inoculated into 96-well plates (4 parasites per well) with HFF cells. After sorting for 7 days, clones were screened through microscope and identified by diagnostic PCRs (F1: GTTCCGCTTCTTTTGTGTCTGCC; R1: GCAGCCTCTTGTATGGCTGT; R2: GATTCCCTCTTTCAGCCCCA).

### 2.6. Immunofluorescent Assay (IFA)

IFA was conducted to evaluate *T. gondii’s* invasion, replication, and egress abilities through marking the parasite’s surface protein. In brief, HFF cells were cultured on coverslips in 24-well plates until confluent, and then they were infected with parasites. During the invasion, replication, and egress assays, at specific designated times, the cells were fixed with 4% paraformaldehyde for 40 min at room temperature (RT). After being washed with PBS, cells were permeabilized with 0.3% Triton TX-100 diluted in phosphate-buffered saline (PBS) for 7 min at RT. The coverslips were blocked by 3% bovine serum albumin (Biosharp, BS114-100 g, Haimen, Jiangsu, China) and incubated consecutively with specific primary and secondary antibodies. Finally, the samples were observed on an LSM980 microscope (Zeiss, Jena, Germany) with a 100×/1.4 oil objective. Images were obtained using a Zeiss Zen Blue 2.3 lite system.

### 2.7. Plaque Assays

Plaque assays were conducted to evaluate the effect of TgRqc2 on parasite growth. HFF cells grown in 12-well plates were inoculated with 700 tachyzoites per well, and the cells were continuously cultured for 11 d. Finally, the cells were fixed with cold methanol and stained by Coomassie Brilliant Blue G250 (Solarbio, Beijing, China).

### 2.8. Invasion Assay

Tachyzoites were added to the monolayers of HFF cells in 12-well plates and allowed to invade for 3 h. Following invasion, the cells were washed with PBS to remove extracellular parasites and then fixed with 4% formaldehyde (Biosharp, Haimen, Jiangsu, China ). Adhered parasites were detected with mouse anti-TgSAG2 antibody and visualized by Alexa Fluor 594-coupled anti-mouse IgG without permeabilization. Subsequently, the cells were permeabilized with 0.3% Triton X-100 in PBS for 7 min at RT. Intracellular parasites were detected using rabbit anti-TgSAG2 antibody and visualized with Alexa Fluor 488-coupled anti-rabbit IgG. At least 100 parasites were counted per slide. This experiment was repeated in three independent biological replicates.

### 2.9. Replication Assay of Intracellular Parasites

Freshly egressed tachyzoites were harvested through sequential extrusion using 27-gauge needles followed by filtration through a 5 μm pore filter. The tachyzoites were then added to monolayers of HFFs in 12-well plates and allowed to invade for 3 h. Extracellular parasites were removed by washing with PBS. Subsequently, the cells were fixed with 4% paraformaldehyde, and the intracellular parasites were labeled using mouse anti-TgSAG2 antibody (1:1000) and visualized with Alexa Fluor 594-coupled anti-mouse IgG (1:1000). The number of parasites per vacuole was determined by counting at least 100 vacuoles per well. The independent experiment was repeated thrice.

### 2.10. Induced Egress Assay

Freshly egressed tachyzoites were harvested and added to monolayers of HFFs in 12-well plates, followed by a 30-h culture period. Subsequently, 3 μM Ca^2+^ ionophore A23187 (C9275-IMG-QM) was added to induce parasite egress. The plate was then incubated in a 37°C water bath for 5 min. Finally, IFA was performed using rabbit anti-SAG2 and mouse anti-SAG2 antibodies to label extracellular (egressed) parasites and intracellular parasites, respectively. At least 100 vacuoles were counted per well. The experiment was carried out with three biological replicates.

### 2.11. Detection of Bradyzoite Cysts In Vivo

The bradyzoite cysts from mouse brains were purified according to previous method with modification [21]. In brief, the brain was washed with PBS and brought up to 1.5 mL in PBS. The brain with suspension was repeatedly passed through a 5 ml syringe needle and 2 mL syringe needle in order until the brain completely turned into brain fluid. Then, the fluid was brought up to a total volume of 2 mL. A total of 0.2 mL fluid was centrifuged, and the pellet was resuspended and fixed using PBS with 4% paraformaldehyde for 30 min at room temperature. An IFA was conducted to detect the cyst where the cyst was stained with Dolichos biflorus agglutinin (DBA)-Fluorescein (Vectorlabs, Newark, CA, USA). Cysts were counted on an EVOS FL auto2 inverted fluorescence microscope using a 20× objective. The results were obtained from three independent biological replicates.

### 2.12. RNA-Seq Analysis

Syringe-released parasites were allowed to invade HFFs in 10-cm dishes for 4 h under normal conditions. Afterward, extracellular parasites were rinsed with PBS and either maintained under standard growth conditions or switched to an alkaline-stress medium. At 48 h post-invasion, parasites were harvested by sequential extrusion through 27-gauge needles followed by filtration through a 5 μm filter, pelleted, and flash-frozen in RNAiso Plus (Takara,Kusatsu, Shiga, Japan). A total of 12 samples (three independent biological replicates per group) were sent to Harbin Haruo Biotechnology Co., LTD. Total RNA was extracted from Trizol. For RNA library construction, polyA-tailed mRNA was enriched from 3 μg of total RNA using Oligo (dT) magnetic beads. First-strand cDNA was synthesized using SuperScript II, followed by second-strand cDNA synthesis employing DNA polymerase I and RNase H. The resulting double-stranded cDNA was purified, and a single “A” nucleotide was added to the 3’ ends prior to adapter ligation. cDNA fragments approximately 400-500 bp in size were selected using AMPure XP beads (Beckman Coulter, Beverly, CA, USA), PCR-amplified, and further purified with AMPure XP beads to generate final libraries. These libraries were then subjected to 150 × 150 paired-end sequencing on the Illumina NovaSeq 6000 platform. Raw sequencing data were processed and filtrated using Fastx-toolkit software version 0.0.13 (http://hannonlab.cshl.edu/fastx_toolkit/index.html). High-quality reads were aligned to the reference genome using Hisat2 [22], and transcript assembly and quantification were performed using StringTie [23]. Gene expression was normalized using FPKM [24] (fragments per kilobase of transcript per million fragments mapped), and this value was used as an indicator to measure the expression level of genes. The differentially expressed genes were analyzed using edgeR software version 4.6.3. [25]. GO enrichment and GSEA enrichment were analyzed using TopGO [26] and KOBAS [27], respectively. Gene sets were considered statistically enriched if the normalized *p* value was <0.05.

## 3. Results

### 3.1. Characterization of RQC Protein TgRqc2

**Figure 1:**
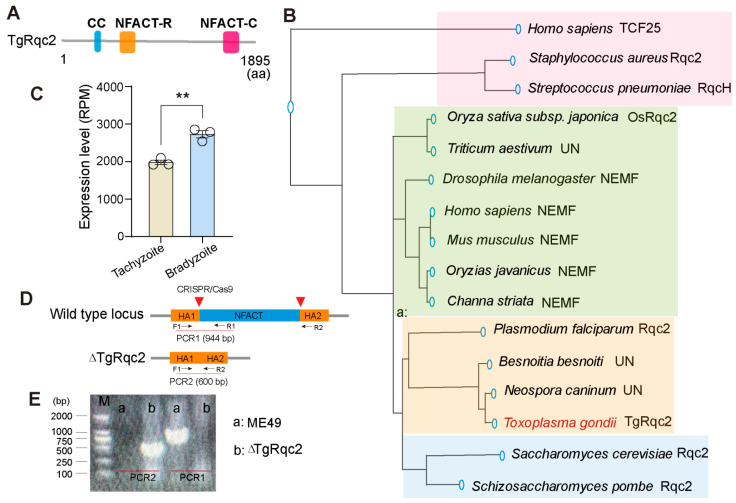
**Identification and characterization of TgRqc2.** (**A**). Schematic structure of NFACT RNA-binding (NFACT-R) and NFACT-C domain. (**B**). Phylogenetic analyses. (**C**). Relative mRNA levels of TgRqc2 in tachyzoite and bradyzoite stages. (**D**). Schematic representation of the CRISPR-Cas9-based system used to deplete TgRqc2. (**E**). PCR analysis of the TgRqc2-knockout strain. NFACT, the “domain” found in the ribosome-associated quality control (RQC) and the complex central players involved in RQC, human NEMF and its orthologs, FbpA (known as RqcH), Caliban, and Tae2 (known as Rqc2). HA, homology arm. F, forward primer, R, revers primer. The data were analyzed by a Student’s t-test. ** *p* < 0.01.

To investigate the potential roles of RQC factors during the growth and development of parasites, we first aimed to identify proteins potentially involved in RQC in *T. gondii*. For this purpose, the peptide sequences of known RQC factor Rqc2 in yeast was used to query the *T. gondii* genome using protein BLAST searches in ToxoDB (https://toxodb.org/toxo/app). The gene with the highest blast score was TGME49_214090. Its structure was further confirmed in UniProt (https://www.uniprot.org/). This protein was found to contain a central NFAT and a coiled-coil region (Figure 1A). As a subunit of the ribosome quality control complex and the ortholog of Rqc2, we named it TgRqc2. Orthologs of Rqc2 from other species were downloaded from UniProt, and phylogenetic analyses revealed that TgRqc2 clustered uniquely with its ampicomplexan orthologs (Figure 1B). While several other proteins showed high blast scores and close evolutionary relationships with Rqc2, they have not been annotated as Rqc2 in ampicomplexan orthologs, such as *Neospora caninum*, *Besnoitia besnoiti*, or in the plant *triticum aestivum*. These were labeled as “unidentified name” (UN) in our phylogenetic analyses. Based on previously published transcriptomic data comparing tachyzoites and bradyzoites [28], we observed a significant increase in the mRNA level of TgRqc2 (Figure 1C). This change in mRNA level suggests that TgRqc2 may play an important role in the differentiation of bradyzoites.

### 3.2. TgRqc2 Is Essential for the Lytic Cycle of T. gondii

**Figure 2:**
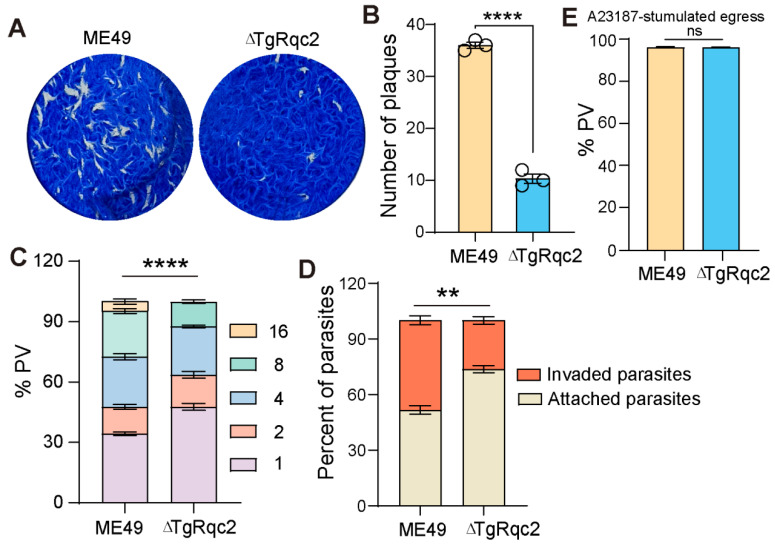
**TgRqc2 is essential for the lytic cycle of *T. gondii*.** (**A**). Plaque assays were performed by infecting HFF cells with WT and ∆TgRqc2 strains. (**B**). Plaque number statistical analysis. (**C**). Parasites after growth for 24 h. (**D**). Invasion assays examining the ability of parasites. (**E**). The egress of parasites from host cells was determined by IFA. The results were obtained from three biological replicates. The data were analyzed by a two-way ANOVA using GraphPad Prism 8 (GraphPad SoftwareInc, San Diego, CA, USA). ** *p* < 0.01, and **** *p* < 0.001.

To further explore the function of TgRqc2 in parasite growth and development, the Rqc2-knockout strain was first constructed using the CRISPR-Cas9 system (Figure 1D) via homologous recombination (HR). The deletion of the gene was confirmed by PCR analysis. The PCR1 fragment was amplified from the wild-type strain, while the PCR2 fragment was amplified from the TgRqc2-knockout strain, indicating the successful deletion of the TgRqc2 gene sequence (Figure 1E). Next, plague assays were conducted using the parental strain and the ∆TgRqc2 strain to evaluate the role of TgRqc2 in parasite growth. The parental strain formed standard plaques after 10 d of culture, while only a few small plaques were observed in the ∆TgRqc2 strain (Figure 2A,B), suggesting a critical role for TgRqc2 in the parasite lytic cycle. To determine which stage of the lytic cycles is affected by TgRqc2 in *T. gondii’s* lytic cycle, a replication assay was conducted. We inoculated wild-type and ∆TgRqc2 strains into fresh HFF monolayers and removed uninvaded parasites 3 h later. The invaded parasites were then cultured for an additional 24 h. The ∆TgRqc2 strain showed significantly reduced proliferation within host cells, with a high percentage of parasitophorous vacuoles (PVs) containing one or two parasites (Figure 2C). An invasion assay was also performed, which revealed that depletion of TgRqc2 significantly impaired the parasite’s ability to invade host cells (Figure 2D). However, the deletion of TgRqc2 did not affect parasite egress ability (Figure 2E). In conclusion, these results indicate that the absence of TgRqc2 severely disrupted the parasite’s normal lytic cycle.

### 3.3. Depletion of TgRqc2 Reduces the Formation of Bradyzoites

**Figure 3:**
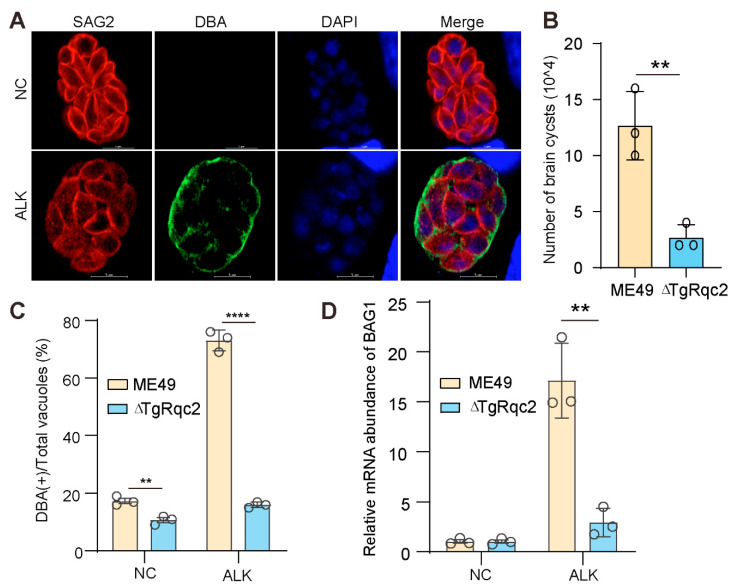
**TgRqc2 is essential for stage conversion**. (**A**). Representative images of *T. gondii* ME49 containing vacuoles exposed to alkaline PH stress for 72 h. DNA was stained with DAPI (blue), the parasitophorous vacuole membrane (PVM) was stained with an anti-SAG2 polyclonal antibody (red), and the cyst tissue was stained with fluorescein-labeled Dohlichos biflorus Agglutinmin (DBA) (green). Scale bar represents 5 μm. (**B**). The detection and quantification of DBA fluorescent staining within all parasites. The total number of vacuoles and cysts was at least 100 for each strain. (**C**). Relative mRNA levels of TgBAG1 in tachyzoite and bradyzoite stages under normal and alkaline stress conditions. (**D**). Quantification of parasites per brain in mice after 4 weeks of infection with ME49 and ∆TgRqc2. The results were obtained from three biological replicates. The data were analyzed by a Student’s t-test using GraphPad Prism 8 (GraphPad SoftwareInc, San Diego, CA, USA). ** *p* < 0.01, and **** *p* < 0.001.

Bradyzoite formation can be induced under alkaline stress conditions. To investigate the potential role of TgRqc2 in bradyzoite differentiation, a bradyzoite induction experiment was performed. Indeed, successful bradyzoite formation was observed under stress culture conditions, as indicated by the contiguous straining of Dolichos biflorus agglutinin (DBA) (Figure 3A). We quantified the bradyzoite conversion rate and found that the efficiency was significantly reduced in the parasites lacking TgRqc2 compared to the wild-type control (Figure 3B). Additionally, both the mRNA levels and the expression of BAG1, a canonical bradyzoites marker, were markedly down-regulated in the absence of TgRqc2 (Figure 3C). Importantly, the role of TgRqc2 in bradyzoite conversion was further validated in vivo. BABL/c mice were infected with the respective parasite strains, and 35 days post-infection, the number of brain cysts was assessed. Consistently, TgRqc2 deficiency impaired bradyzoite formation in the brain (Figure 3D). Collectively, these results demonstrate the critical role of TgRqc2 in bradyzoite conversion.

### 3.4. Depletion of TgRqc2 Reprograms the Transcript Profile

**Figure 4:**
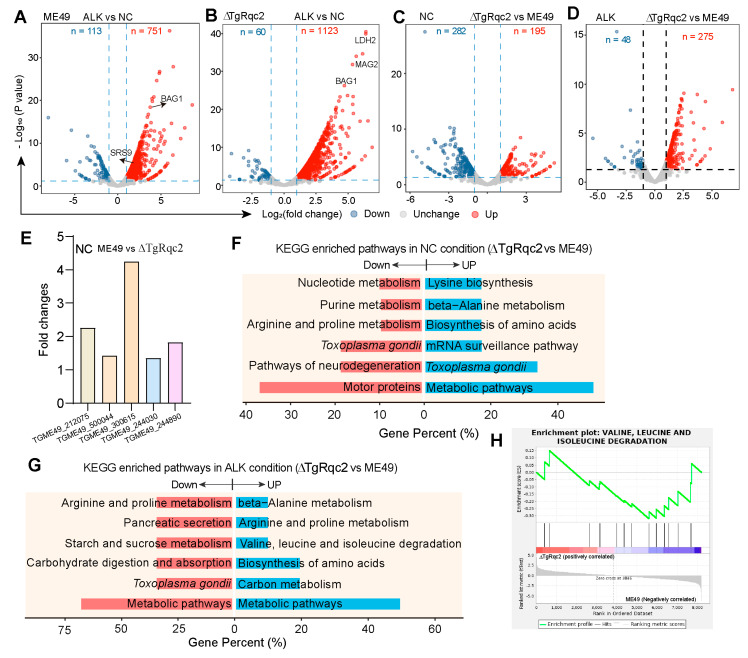
**Transcriptomic analysis of gene expression in parasites under normal and alkaline stress conditions.** (**A**–**D**). Volcano maps of differently expressed genes between Me49 and ∆TgRqc2 parasites. (**E**). Specific genes downregulated after the deletion of TgRqc2 under normal condition. (**F**,**G**). KEGG analysis of differently expressed genes between Me49 and ∆TgRqc2 groups. (**H**). GSEA enrichment analysis between Me49 and ∆TgRqc2 groups under normal condition. Three biological replicates were used in each group.

The accumulation of ribosome stalling products can lead to prematurely polyadenylated non-stop mRNA translation in eukaryotes. Additionally, the poly(A) tail-encoded poly-Lys tract can function as a nuclear localization signal that engages the nuclear transport machinery [29], leading to accumulation in the nucleoli and the disruption of transcriptional homeostasis [30]. To assess the impact of TgRqc2 on parasite transcription, transcriptomic sequencing was performed on *T. gondii* collected under normal and alkaline conditions. Regardless of whether the strain was the wild-type or the ∆TgRqc2-knockout strain, the mRNA levels of bradyzoite stage-specific genes, such as BAG1, SRS9, and LDH2, were upregulated under stress conditions compared to normal conditions (Figure 4A-B), indicating a conversion of tachyzoites into bradyzoites. As expected, the deletion of Rqc2 caused more pronounced transcriptomic changes, with 1123 genes upregulated compared to the wild-type strain under alkaline conditions (Figure 4B). The depletion of TgRqc2 resulted in the downregulation of 282 genes and the upregulation of 195 genes under normal culture conditions, while 48 genes were downregulated and 275 genes were upregulated in response to alkaline induction (Figure 4C-D). Among the genes altered under normal conditions, several specific genes involved in the parasite lytic cycle were significantly downregulated, including meiosis-specific nuclear structural protein 1 (TGME49_212075) (Figure 4E). Pathway analysis of differentially expressed genes revealed abnormal amino acid metabolism in both conditions following TgRqc2 deletion (Figure 4F,G). Moreover, GSEA enrichment analysis showed the activation of the amino acid metabolism pathway after TgRqc2 deletion, suggesting its potential role in regulating translation.

## 4. Discussion

The RQC pathway is activated when translation elongation kinetics are disrupted or when the ribosome translates the polyA tail of non-stop mRNA [31]. The dysregulation of the RQC process can be toxic to cells. While it is well-established that the conversion of *T. gondii* from the tachyzoite to the bradyzoite stage occurs under stress conditions, and that significant changes in protein expression and translational regulation accompany this transition, no studies have yet investigated the role or function of RQC in *T. gondii*.

In this study, we characterized the RQC component TgRqc2 and elucidated its role in the lytic cycle and stage conversion of the parasite. We confirmed that TgRqc2 contains an NFACT RNA-binding domain that exhibits selectivity for tRNAs to generate the C-terminal tails of nascent polypeptide chains. The phylogenetic analysis of RQC components from bacteria to humans revealed four major branches, with TgRqc2 forming a distinct clade alongside orthologs from other apicomplexan species. The depletion of TgRqc2 significantly impaired plaque formation, underscoring its essential role in the parasite lytic cycle. These findings indicate that the RQC pathway is crucial for parasite survival. RNA-Seq analysis showed that deletion of TgRqc2 led to transcriptomic alterations, particularly affecting genes involved in *T. gondii’s* life cycle. These gene expression changes likely contribute to the growth defect observed in ∆TgRqc2 parasites. A recent study identified ubiquitin-activating enzyme1 as a key regulator of the *T. gondii* lytic cycle and cellular homeostasis [32]. Rqc2 has been shown to recruit ubiquitin ligase to the ribosome complex to facilitate protein degradation [32,33]. However, whether TgRqc2 participates in the parasite lytic cycle through interaction with ubiquitin ligase remains to be determined.

The accumulation of ribosome stalling products can lead to the translation of prematurely polyadenylated non-stop mRNA in eukaryotes. The poly(A) tail-encoded poly-Lys tract can act as a nuclear localization signal and engage the nuclear transport machinery [29], leading to accumulation in the nucleoi and the disruption of transcriptional homeostasis [30]. Moreover, ribosome stalling can slow down translation during the decoding of poly-Lys sequences encoded by “AAA” codes [34]. RNA-seq analysis revealed significant reprogramming of transcriptional profiles. Pathway analysis indicated abnormal amino acid metabolism, which may have impacted the proteins essential for parasite growth. However, this study did not include experiments to confirm the accumulation of the poly(A) tail-encoded poly-Lys tract. In our future work, proteomics analysis and ribosome profiling will be performed to elucidate the changes in translational control.

The conversion of fast-growing tachyzoites into slow-growing bradyzoites is a critical factor in establishing chronic infection, enabling long-term persistence, and ultimately facilitating transmission to the next host [5,35]. Our results highlight the essential role of TgRqc2 in parasite development. Similar to other known methods of inducing stage conversion—such as exposure to acidic conditions or heat shock—these approaches generally involve subjecting the parasites to stress [3]. In our study, we successfully induced bradyzoite formation using an alkaline medium. The translational machinery is closely linked to environmental conditions, making ribosomes ideal candidates for sensing cellular states and serving as platforms for various signaling pathways in response to cellular changes. Ribosomes typically slow down and stall in response to stress [12]. Therefore, we hypothesize that TgRqc2 plays a role in regulating bradyzoite formation through the RQC pathway. Although our understanding of the components and mechanisms involved in RQC has expanded, how this process is regulated by cellular signaling pathways in parasites remains poorly understood. Further research is needed to elucidate the underlying regulatory mechanisms.

In recent years, the regulatory mechanism of RQC has been extensively studied in amyotrophic lateral sclerosis, Parkinson’s disease, Huntington’s disease, and Alzheimer’s disease [36,37,38,39]. Developing drugs that target RQC for the treatment of neurodegenerative diseases holds significant advantages, as RQC is an inherent cellular surveillance and clearance system already present in cells [40]. Although no RQC-targeting drugs have been reported to date, compared to other novel therapeutic approaches, targeting RQC may potentially reduce the likelihood of adverse side effects. Similarly, given the essential role of Rqc2 in parasite growth, it represents a highly promising potential target for the treatment of toxoplasmosis.

In conclusion, our data demonstrate that TgRqc2 is a key determinant of *T. gondii* persistence, owing to its essential role in the parasite lytic cycle and bradyzoite conversion. This discovery raises intriguing new questions about the regulation mechanisms of TgRqc2 that influence parasite growth and stage transition. Further investigation into RQC is likely to reveal critical insights into the commitment to differentiation. Collectively, our findings will contribute to the development of therapeutic strategies aimed at preventing chronic infection.

## Data Availability

The original contributions presented in this study are included in the article. Further inquiries can be directed to the corresponding authors.
